# Introducing a novel model for simulating large loop excision of the transformation zone (LLETZ) using 3D printing technique

**DOI:** 10.1007/s00404-021-06209-1

**Published:** 2021-09-07

**Authors:** Matthias Kiesel, Inga Beyers, Adam Kalisz, Achim Wöckel, Saskia-Laureen Herbert, Carolin Curtaz, Joachim Diessner, Ralf Joukhadar, Christine Wulff

**Affiliations:** 1 Department of Gynecology, University Hospital Würzburg, Josef-Schneider-Str. 4, 97080 Würzburg, Germany; 2 Institute of Electric Power Systems (IfES), Leibniz Universität Hannover, Appelstraße 9A, 30167 Hannover, Germany; 3 Department of Electrical, Electronic and Communication Engineering, Information Technology (LIKE), Friedrich-Alexander-Universität Erlangen-Nürnberg, Am Wolfsmantel 33, Erlangen, Germany

**Keywords:** 3D printing, Virtual, Gynecology, Dysplasia, Large loop excision of the transformation zone (LLETZ), Fused deposition modeling (FDM)

## Abstract

**Purpose:**

Electrosurgery is the gold-standard procedure for the treatment of cervical dysplasia. The quality of the outcome depends on the accuracy of performance, which underlines the role of adequate training of surgeons, especially, as this procedure is often performed by novice surgeons. According to our knowledge, medical simulation has up until now lacked a model, which focuses on realistically simulating the treatment of cervical dysplasia with the concerning anatomy.

**Methods and result:**

In our work, we present a model created using 3D printing for holistically simulating diagnostic, as well as surgical interventions of the cervix, as realistically as possible.

**Conclusion:**

This novel simulator is compared to an existing model and both are evaluated. By doing so, we aim to provide novice gynecologists with standardized and high-quality simulation models for practicing to improve their proficiency.

## Introduction

Cervical cancer poses a considerable threat to women around the world. From a global perspective, it shows the fourth highest incidence as well as mortality of cancers among women [1]. The majority of cervical cancer originates from the dysplasia of cervical cells. Compared to actual cancer, the incidence of cervical dysplasia is around one hundred times higher. The frequency of new cases of such precancerosis lays around 1% of all women [2]. Thanks to the screening with Pap-Smear, which normally contains cells of the transformation zone, cases of cervical precancerosis can be detected [3]. If the cytological results indicate the risk of dysplasia, the cervix can be examined via colposcopy by primarily applying acetic acid. This can potentially be followed by Lugol’s iodine for further evaluation [4, 5]. If colposcopy also shows signs of dysplasia, a selective biopsy can be taken. In case of a High-Grade Squamous Intraepithelial Lesion (HSIL), therapy can be indicated. It is the aim of the local therapy to remove dysplastic areas as well as the transformation zone of the cervix (R0-resection) [6–9]. This can be done by performing a so-called large loop excision of the transformation zone (LLETZ) [2, 10]. By doing so, caution has to be taken not to remove too much tissue of the cervix, as it is possibly required for future pregnancies of the patient. Consequently, excessive resection has to be avoided, since it can lead to preterm birth afterwards. In addition, surgical interventions concerning the cervix can lead to scarring. This can prevent the future ascension of sperm, leading to infertility of the patient [11–18]. Consequently, especially for patients with not yet completed family planning, a carefully and professionally performed resection is pivotal [19]. Whereas some surgical techniques can only be trained in the operation room, it is often possible to simulate some techniques so that model-training can be performed to improve the technique, thus enhancing patients’ safety [20–26]. Unfortunately, in many cases sufficient training is hampered by the organizational effort required and a lack of personnel and time in everyday clinical practice. Additionally, there are no standardized simulators for procedures such as the LLETZ. Yet possible ideas for such simulators or phantoms can be found in many parts of literature [20, 21, 23–30]. Often rather simple simulators are used or produced, which are easily and quickly assembled and do not require too many financial resources. In many cases, materials such as sponges, sausages and cardboard boxes are used [21, 26, 29]. We reviewed the existing literature and found the existing models insufficiently represent the real anatomy. According to our knowledge, medical simulation has up until now lacked a model, which focuses on realistically simulating the treatment of cervical dysplasia with the concerning anatomy.

## Aims and questions

In our work we wanted to create and describe a novel approach of simulation for the procedure of LLETZ. Our focus should be on simulating standardized diagnostic and surgical steps of treatment of dysplasia of the cervix as realistically as possible. We also wanted to compare this novel approach to existing simulators, to improve simulation quality and training variation. By doing so, we aim to support the proficiency of novice surgeons and consequently the safety of our patients.

## Methods and material

**Fig. 1:**
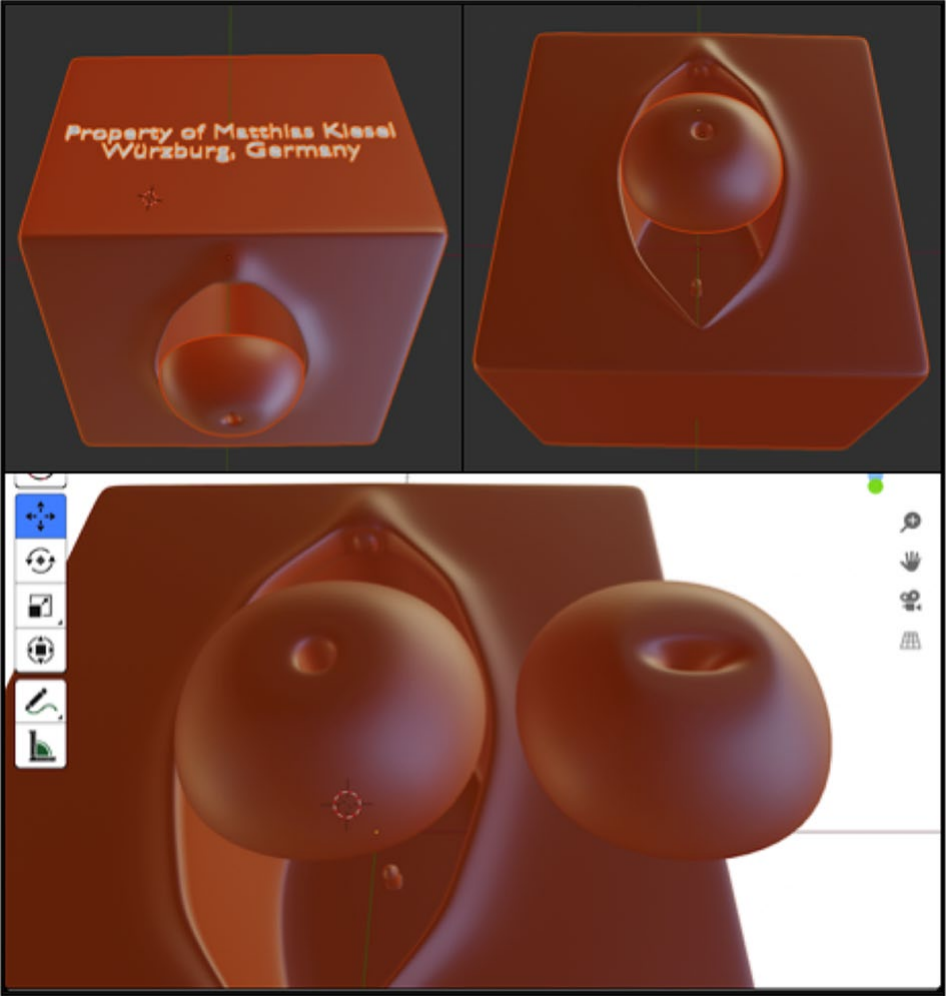
Digital 3D modeling of the vulva, vagina and cervix (uni-/multiparous) using *Blender* version 2.82

We used the open-source-program *Blender*, version 2.82 for producing a virtual model of a vulva and vagina. The cervix, available as nulli- or multiparous, was sculptured as a separate object. This is depicted in Fig. 1. These models were produced in a realistic size by Fused Deposition Modeling (FDM), using a 3D-printer (*Ultimaker 2* + *, used filament: Polyactide (PLA), 2.85 mm, from the company „DAS FILAMENT “, Article number F10255*). By doing so, a cube made of plastic was produced, incorporating a life-sized, vagina-shaped deepening, in which the model of the cervix can be inserted.

For the sake of reassembling natural tissue, we duplicated these models by using modeling silicone. In a first step, we coated the 3D-printed vagina with silicon mold separating cream (*Troll Factory (TFC), Article number: TFC3101*). It was then placed in an equally coated box, which was filled with modeling silicone (*Abform-Silikon, Laurenz* + *Morgan GmbH, Article number: 1000634*). As soon as the silicone had hardened, we removed the 3D-printed vagina, leaving a negative footprint on the inside of the silicone-cube.

**Fig. 2:**
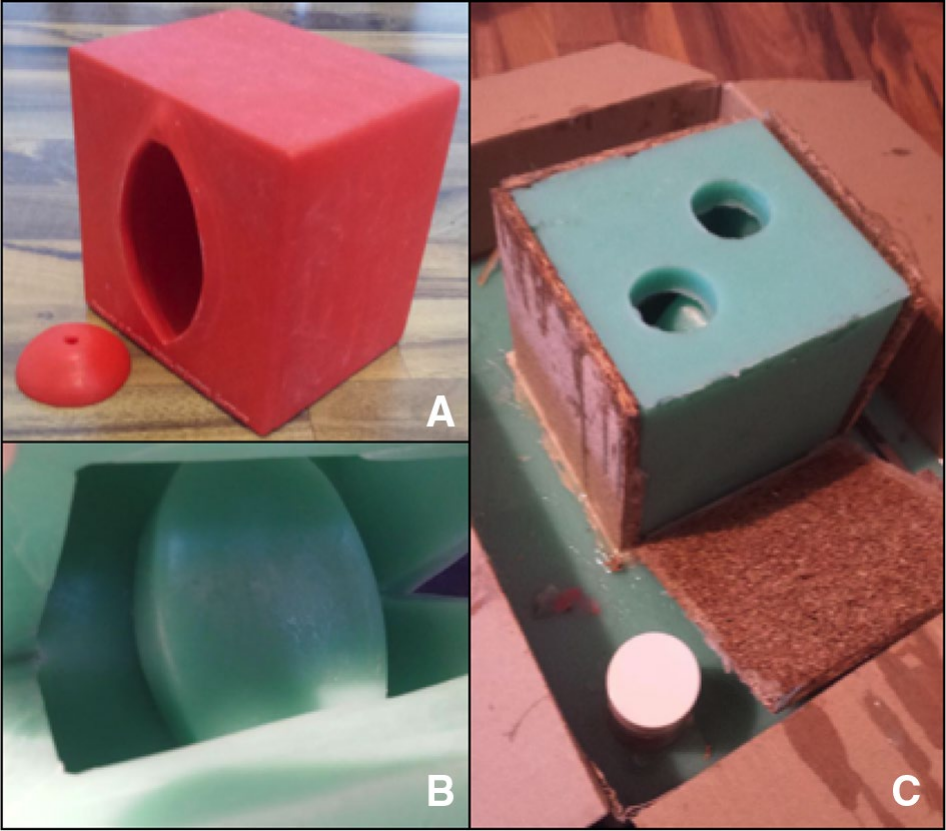
Construction of the simulator. **A** By using Fused Deposition Modeling (FDM) 3D printing, we created an artificial vulva, vagina und cervix, **B** NEgative footprint made of silicone, looking into the inside of the mold, **C** the mold from the outside, ready to be filled with silicone

This negative footprint was again coated with silicon mold separating cream and then filled with modeling silicone. In this case, we used *Wagnersil 22 NF* of the company *Wagner Dental GmbH & Co. KG (Article number: 42165100*). After having hardened, the silicone was removed from the silicone-cube. Now we had created a silicone-copy of the initially 3D-printed model of the vagina. To create a certain variation of training and to reassemble the natural anatomy more correctly, we additionally built a second version with a deeper and narrower vagina. Exemplary pictures of the production process can be seen in Fig. 2.

**Fig. 3:**
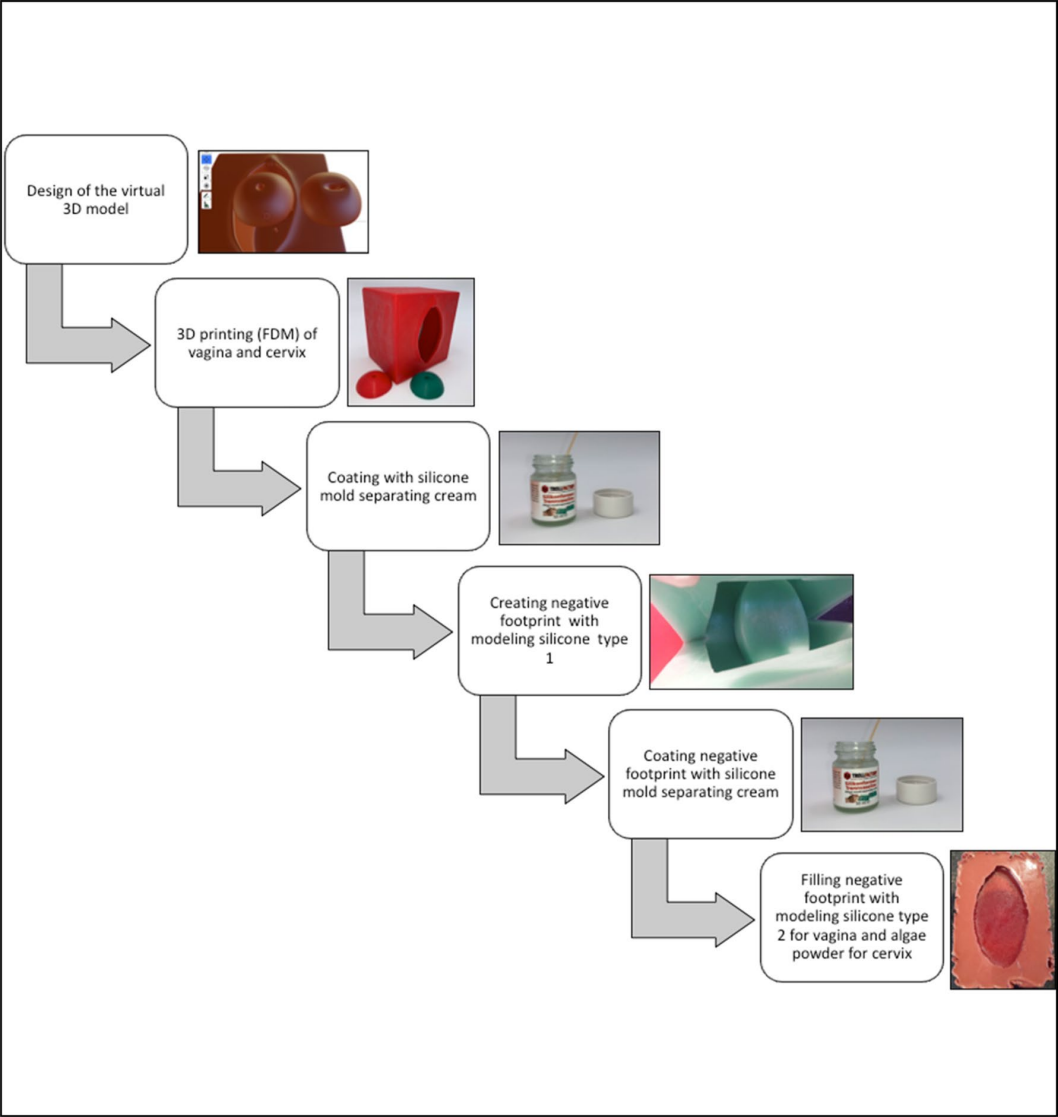
Simplified flowchart of the production process of the described simulator: Silicon mold separating cream: *Abform-Silikon, Laurenz* + *Morgan GmbH, Article number: 1000634.* Modeling silicone type 1: *Abform-Silikon, Laurenz* + *Morgan GmbH, Article number: 1000634* Modeling silicone type 2: *Wagnersil 22 NF, Wagner Dental GmbH & Co. KG (Article number: 42165100*)

**Fig. 4:**
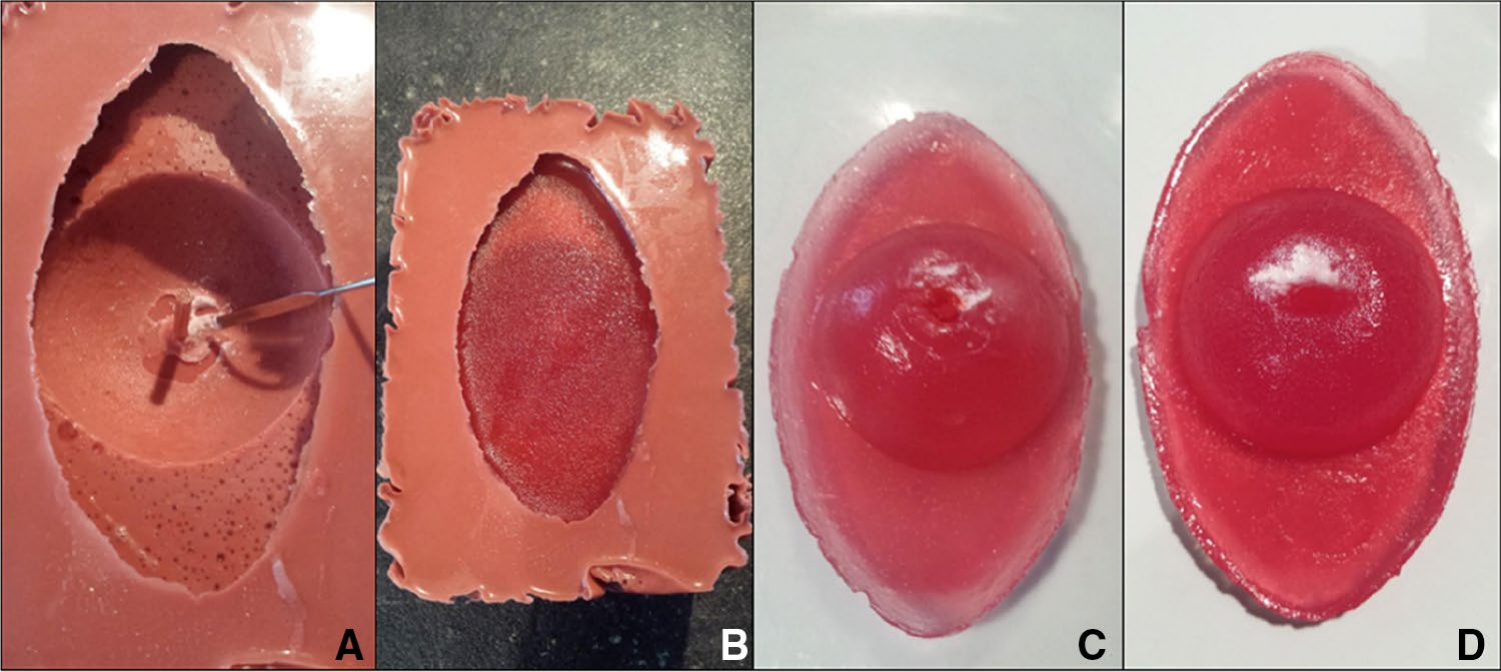
Production of the cervix-model. **A** Mold with negative footprint made of silicone. Agar–Agar-powder mixed with water and white food coloring is being applied with a spatula. For adding more depth to the cervical canal, a wooden stick is integrated in the middle of the mold, **B** mold after hardening of the Agar–Agar-mixture, **C** nulliparous cervix-model with imitation of transformation zone and dysplasia after being removed from the mold, **D** multiparous cervix-model with imitation of transformation zone and dysplasia

The model of the cervix was duplicated in a similar manner. Yet in this case, we did not use silicone to fill the negative footprint. Instead, we filled it with customary Agar–Agar (*Agartine, RUF Lebensmittelwerk KG*). Agartine is a plant-based gelling agent, which is produced from algae. It was dissolved in water, heated and then filled in the silicone negative footprint of the cervix to harden. This enables us to create a copy of the 3D-printed cervix model made out of algae powder. Using the correct proportion of water and Agar–Agar, the cervix is sufficiently dimensionally stable and fits into the silicone model of the vagina. According to our experience, 10 g of Agartine-powder, being brought to boil together with 60 ml of tap water for few seconds, lead to adequate results after hardening. Mixing red food coloring (*Back- und Speisefarbe, rot, Rosenheimer Gourmet Manufaktur GmbH*) to the tap water adds to a more realistic appearance. A transformation zone can be imitated by applying single drops of red food coloring around the entrance to the cervical canal of the hardened Agartine-cervix. White food coloring (e.g. *White-White Icing Colour, Wilton Industries, INC*), mixed with algae powder, can simulate the stains of abnormal areas after applying acetic acid on the cervix. After hardening of the Agar–Agar, the now created artificial cervix shows areas of white stains similar to abnormal areas on a cervix, which has been washed with acetic acid. A simplified flowchart of the production process can be seen in Fig. 3. These Agar–Agar-models for the cervix can be kept refrigerated for several weeks. They can also be frozen and are ready to use after thawing. Further pictures can be seen in Fig. 4.

**Fig. 5:**
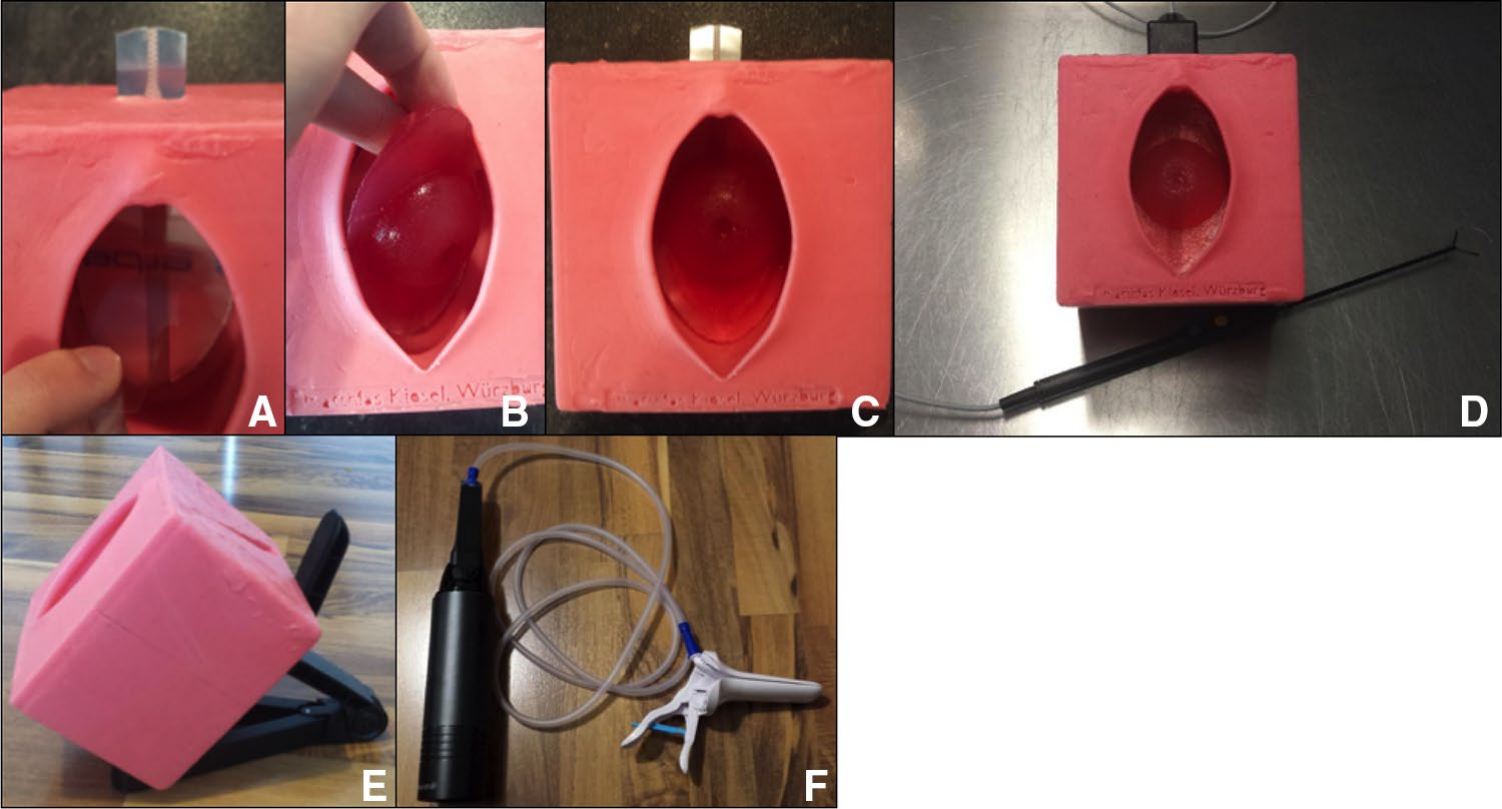
Preparing the simulator. **A** Applying the neutral electrode, **B** inserting the model of the cervix, C: Cervix with transformation zone without dysplasia, **D** simulator connected to neutral electrode and electrosurgical loop (company: *Erbe*), **D** positioning the simulator on a tablet-holder, **E** handheld vacuum cleaner (company: *LifeBasis*) connected to speculum (company: *Bridea Medical*) for smoke gas retraction

In the next step, we fitted a customary neutral electrode (*ERBE NESSY*^*®*^* Omega Plate*, *Erbe Elektromedizin GmbH, Article number 20193–082)*, which is also used for real LLETZ-procedures, into the silicone model of the vagina. Now the Agar–Agar-model of the cervix can be placed on top of the adhesive part of the electrode. The cable of the electrode is connected to the generator of the electrosurgical loop (*Erbe, VIO® 300D, Article number: 10140–100)*. Respective pictures can be seen in Fig. 5.

After this preparation, the simulator is now ready for use. The silicone cube containing the model of the vagina can be placed on e.g. a gynecological chair, a desk etc. and a speculum can be inserted into the artificial vagina. When using a flat surface such as a table, which cannot be tilted, we found that a simple tablet-holder for handheld tablet computers (*PEARL Tablethalter, Modell SD-2200–675, PEARL.GmbH*, Fig. 5) can sufficiently simulate the angle of the female pelvis during colposcopy.

If the electrosurgical device (such as *Wolfram Schlingenelektrode, Erbe Elektromedizin GmbH, e.g. Article number 21191–045*) is activated and touches the Agar–Agar-cervix, the cervix conducts electricity such as real tissue. Consequently, the circuit closes and the model of the cervix reacts to electrosurgery equal to a human cervix. The produced smoke can be removed using a speculum with integrated smoke gas extraction (e.g. *Orchid Wide SX, Bridea Medical, Article number: 1.031.44*). As we wanted to make training more flexible and independent, we built a portable device for removing gas by attaching a handheld, battery operated vacuum cleaner (*LVC-101-EB* from the company *LifeBasis*) to a single-use gas-extraction-hose, which is compatible with the used speculum. Hence, a stationary surgical customary device for smoke gas extraction is no longer needed. As a consequence, only a colposcope and an electrosurgical device for LLETZ-procedure are required from the hospital’s resources. This described simulator shall be called “novel simulator” in the following.

On the basis of the work of Takacs et. al., Connor et. al. and Walters et. al. we constructed another simulator for LLETZ-procedure, built from easily accessible material [20, 21, 28, 31]. Several authors have already shown that good learning effects can be reached with such simple devices [20, 21, 26–28]. Consequently, this known type of simulator shall be referred to as “conventional” simulator. We used a t-shaped drain pipe made from Polypropylene (*Ostendorf Kunststoffe GmbH,* purchased via *HORNBACH Holding AG & Co. KGaA, Article number 266386*) and inserted pipe insulation material made of polyethylene (*NMC Deutschland GmbH*, purchased via *HORNBACH Holding AG & Co. KGaA, Article number: 7625278*). This construction should simulate the vagina. For the sake of stability, it was glued onto a plastic board. As sausage, reassembling the cervix, we chose *Curryfleischwurst, Meister feines Fleisch – feine Wurst GmbH*. The same customary neutral electrode (*ERBE NESSY® Omega Plate*, *Erbe Elektromedizin GmbH, Article number 20193–082)*, attached to the sausage, and speculum for smoke gas retraction (*Orchid Wide SX, Bridea Medical, Article number: 1.031.44)* was used as with the novel simulator*.* Pictures of the used conventional simulator can be seen in Fig. 12.

## Results

**Fig. 6:**
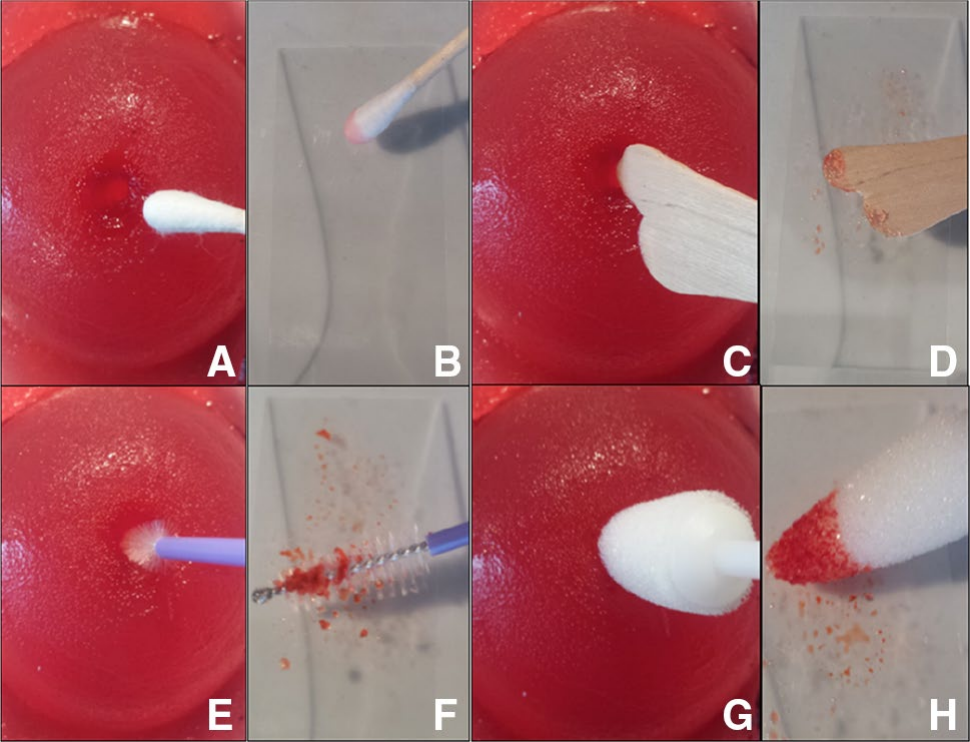
Pap-smear. **A**, **B** Regular swab, **C**, **D** wooden spatula, **E**, **F** brush for endocervical smear, **G**, **H** conical swab for Pap-smear of pregnant patients

**Fig. 7:**
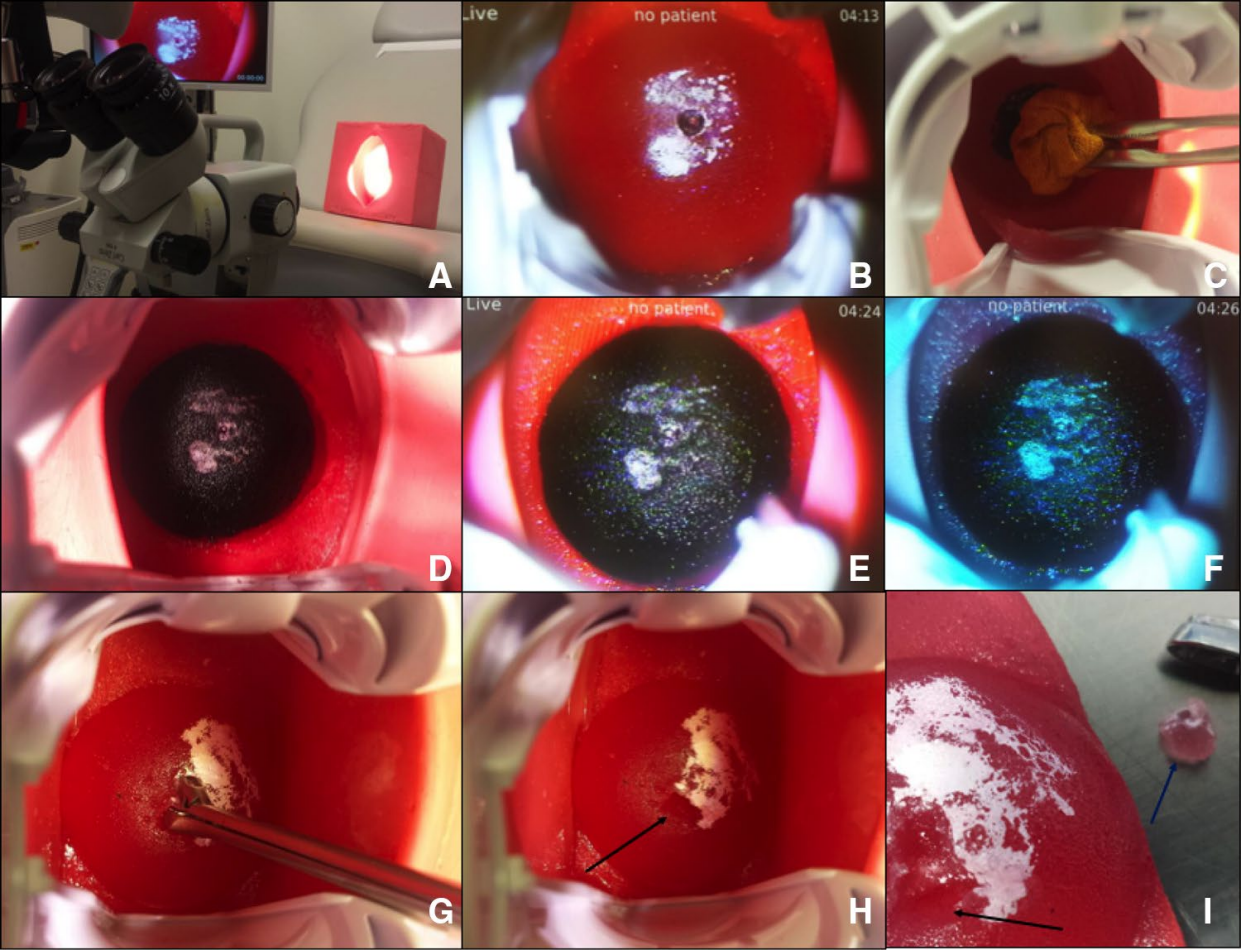
Colposcopy, Lugol’s iodine and biopsy. **A** Placing the simulator on a gynecological chair with attached colposcope (company: *ZEISS*), **B** artificial cervix with imitation of dysplasia (colposcopic view). A speculum of the model *Orchid Open* from the company *Bridea Medical* is used, **C** applying Lugol’s iodine on the cervix, **D** cervix after applying Lugol’s iodine: regular areas appear dark and abnormal areas remain white, **E** colposcopic view after applying Lugol’s iodine, **F** colposcopic view using a green filter, **G** preparing the biopsy forceps, **H** arrow marking the area after biopsy, **I** cervix-model after being removed from the artificial vagina with tip of biopsy forceps (black arrow marking area of biopsy, blue arrow marking removed tissue). **G**, **H** Show the variation of the artificial vagina with less width and more depth

While testing the new simulator’s functionality, we found it can be used to imitate different procedures in addition to the LLETZ-procedure itself: Firstly, a regular Pap-smear can be accomplished. Due to the structure of the hardened Agar–Agar, particles are peeled off, remain attached to the swab and can then be applied on the slide of the microscope. This works for the surface of the cervix as well as the cervical canal, which is shown in Fig. 6. Secondly, the artificial cervix can be dabbed with Lugol’s iodine, turning the surface brown, equal to normal squamous epithelium of a real cervix. The white areas marked with white food color remain visible, thus appearing like dysplastic areas in reality after washing with acetic acid followed by iodine. Moreover, cervical biopsies can also be taken from these areas using a biopsy forceps. This is depicted in Fig. 7.

**Fig. 8:**
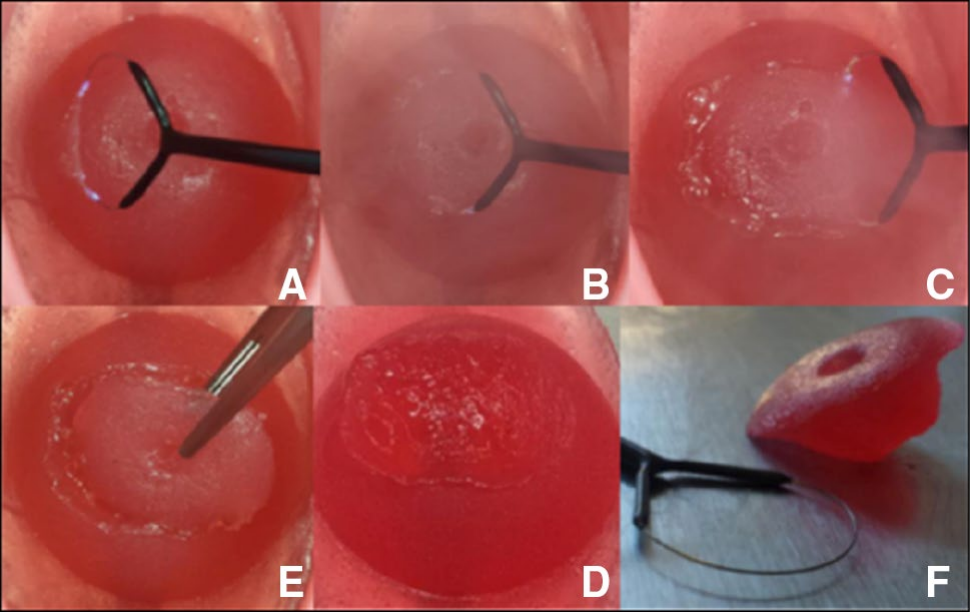
Performing the procedure of LLETZ (artificial cervix without dysplasia). **A**–**C** Preparation of the cone with electrosurgical loop. **E** Removing the cone with tweezers, **D** artificial wound surface after conisation. **F** Cone together with electrosurgical loop

**Fig. 9:**
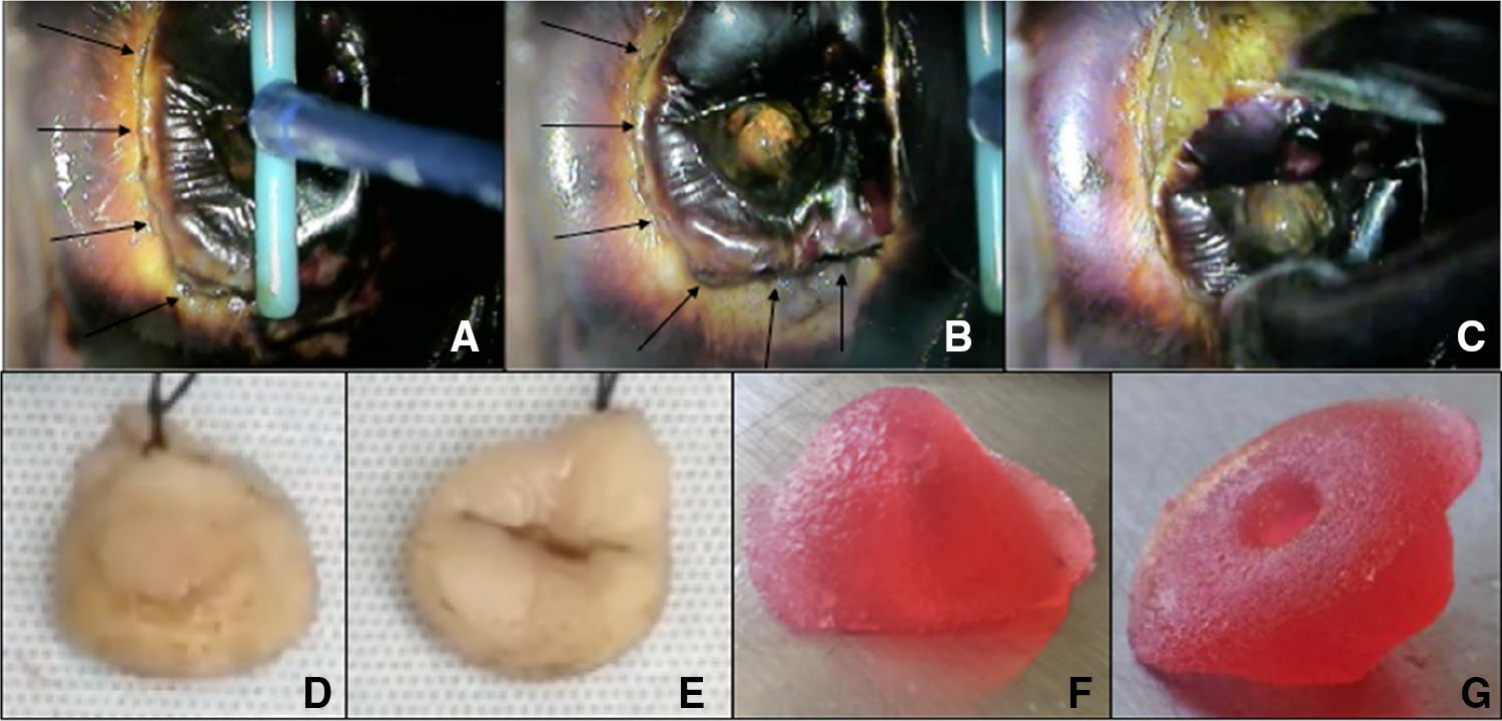
Performing the procedure of LLETZ (real portio). **A**–**C** Conisation of a real cervix after applying Lugol’s iodine, arrows marking the path of the electrosurgical loop, **C** cone being removed with a forceps [32]. **D**, **E** Real cone with thread (no application of iodine), **D** Cone from the backside (endocervical), **E** Front of cone (ectocervical) [33]. **F**, **G** Show the backside and front of the artificial cone

**Fig. 10:**
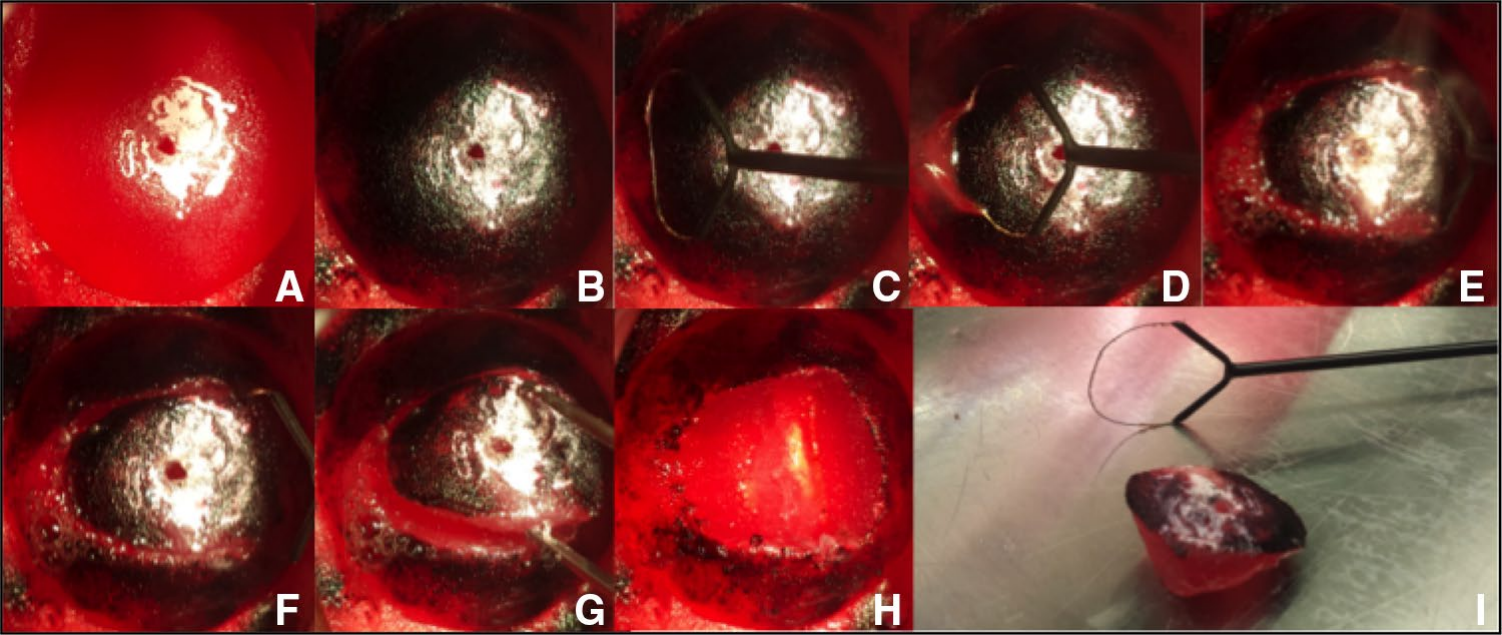
Performing the procedure of LLETZ (artificial cervix with dysplasia). **A** Cervix with imitation of stains after acetic acid, **B** Cervix after application of Lugol’s iodine, **C**–**F** procedure of LLETZ, **G** Cone being removed with forceps, **H** artificial wound surface after conisation, **I** Cone together with electrosurgical loop

**Fig. 11:**
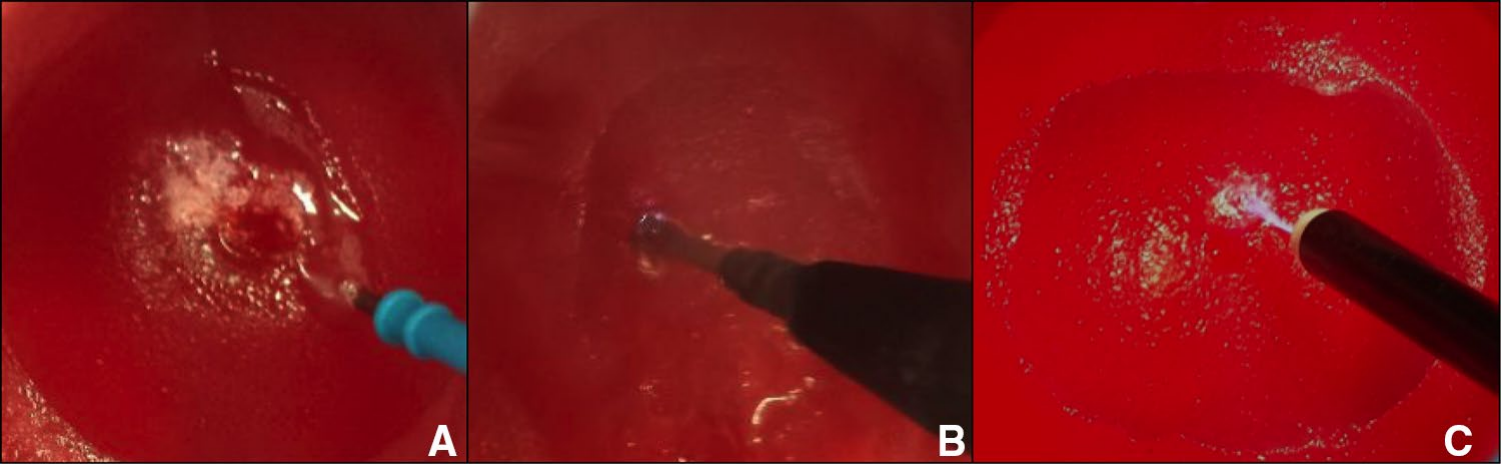
**A** Cutting the artificial cervix using a monopolar knife (*VIO, ERBE, Article number: 21191–459*), **B** Coagulation of the wound surface using a monopolar ball electrode (*VIO, ERBE, Article number: 21191–364*), **C** Coagulation of the wound surface using a device for argonplasma-coagulation (*VIO, ERBE, Article number: 20132–032*)

**Fig. 12:**
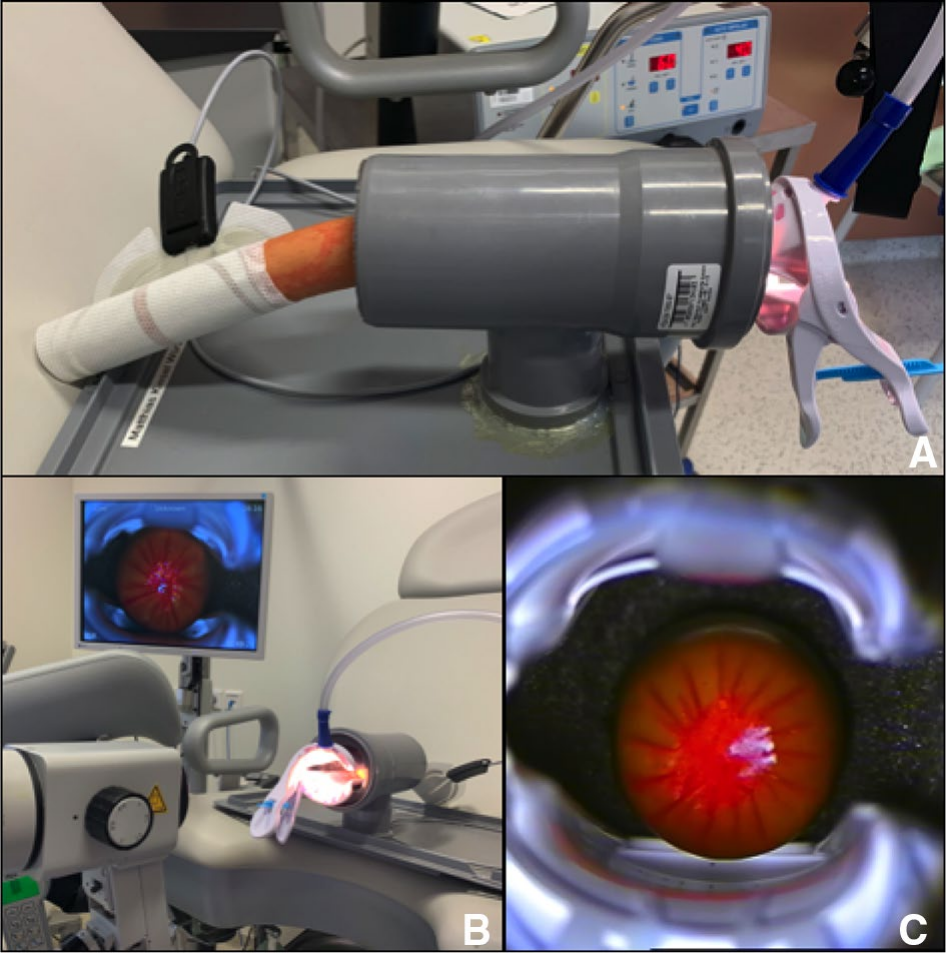
**A**–**C** Conventional simulator for LLETZ. Sausage and speculum are inserted into the drain pipe fitted with the insulation material. The end of the sausage, hanging out of the drain pipe, is connected to the neutral electrode. **B** The monitor in the upper left corner of the picture shows the colposcopic view of the viedo-colposcope. **C** Colposcopic view: The sausage (*Curryfleischwurst, Meister feines Fleisch – feine Wurst GmbH*) is painted with red food color (*Back- und Speisefarbe, rot* from *Rosenheimer Gourmet Manufaktur GmbH*), in order to simulate the T-Zone as well white food color (*White-White Icing Colour, Wilton Industries, INC*), in order to depict dysplasia of the cervix

For the sake of illustration, Fig. 8 shows the process of the LLETZ-procedure using the novel simulator without abnormal findings. Figure 9 depicts a LLETZ in reality. Furthermore, artificial and real results of the surgical preparation are shown. Adding to this, Fig. 10 illustrates the LLETZ-procedure using the novel simulator, imitating abnormal findings after the application of acetic acid and Lugol’s iodine. We found the novel simulator can also be used with a ball electrode (*Erbe Elektromedizin GmbH, e.g. Article number 21191–124)*, an argon-plasma-coagulation-device (*Erbe Elektromedizin GmbH, e.g. Article number 20132–201)* and a monopolar knife (*Erbe Elektromedizin GmbH, e.g. Article number 21191–459*), as seen in Fig. 11. After the training, the model of the cervix can be replaced. The neutral electrode can be reused several times (see Fig. 12).

## Discussion

Our presented simulator for training surgery such as the LLETZ of the cervix is a new approach for training of novice gynecological surgeons. Until now, simulators for LLETZ-procedures mostly used simple materials, which only partly resembled the real anatomy of the cervix [20, 21, 23–29]. For instance, Reeves et. al., Rezniczek et. al., Hefler et. al., Wilson et. al. Walters et. al., Takacs et. al. and Seltzer et. al. could show that using a sausage to imitate the cervix can be of use for training electrosurgical excisions of the transformation zone [20, 23–27, 31]. Manley et. al. created a simulator for the training of cervical biopsies by using a sponge [29]. Until now, simulators are lacking, which display as many parts of a dysplasia consultation as possible and secondly do so in a standardized manner. Additionally, the existing simulators represent the real anatomy insufficiently.

Comparing both simulators, novel and conventional, reviewing the process of the LLETZ step by step, we found that firstly, the novel simulator represents the real picture of a cervix more closely than the conventional simulator using a sausage. Secondly, the broader surface of the novel cervix enables the surgeon to choose from the entire variety of electrical loop-sizes, according to the size of the dysplastic areas, thus making the novel simulation more realistic. We found that the sausage of the conventional simulator was too small for adequately enabling a LLETZ with a loop of 20–25 mm diameter. Thirdly, the two variants of the vagina of the new simulator make it possible to vary within depth and width, whereas the conventional simulator may only vary within the simulated vaginal depth. In addition, we could further increase the possibilities of variation with the novel simulator by creating two different types of cervix (nulli- and multiparous). Moreover, the novel simulator shows the cervical canal after performing the LLETZ, as it contains a real canal reaching into the depth of the artificial tissue. This enables the surgeon to perform a precise second excision, in order to receive a second cone. In reality, this can be necessary, if e.g. the surgeon realizes that his/her cone was too flat for R0-resection, or if dysplastic cells were diagnosed reaching down into the cervical canal beforehand. This possibility of a second, deeper resection is also offered by the conventional simulator, but the second cone has to be resected more or less blindly, as the conventional simulator does not show a cervical canal.

Furthermore, during the LLETZ the conventional simulator produced more smoke than the novel simulator. On the one hand, this makes the conventional simulator more realistic. On the other hand, even with sufficient smoke gas extraction, it makes visualization during the LLETZ more difficult for an inexperienced surgeon. After several simulations, we found that the adhesive part of the neutral electrode began to dissolve due to the moisture of the Agar–Agar-formed cervix. This increases the demand for neutral electrodes over time. Yet, while removing the electrode from a used sausage, we made the experience that parts of it stuck to the electrode, making it necessary to renew it over time as well.

Nevertheless, the novel simulator also showed inferiority in some aspects, compared to the conventional simulator. Firstly, the cone after LLETZ-procedure is more fragile, since the algae-powder seems to soften slightly when heated by the electrosurgical loop. This can lead to damaging the cone when removing it imprudently from the cervix with tweezers. The cone of the conventional simulator appeared sturdier and could also be removed easily with pointed tweezers. Secondly, economically speaking, the purchased Agar–Agar for one novel cervix had a price of 33 cents. The type of sausage we used had a price of 1 € each. Although this makes the use of the novel simulator less price-intensive than the conventional simulator, this advantage is outweighed by the more resource-intensive production of the novel simulator. For instance, the pure material costs for the novel simulator are 263.11 €. The costs for the individual components are summarized in Table 1.

**Table 1:** Production costs for novel simulator

Material	Production costs
3D printed model of vulva and vagina	14.24 €
3D printed model of cervix (nulliparous)	1.56 €
3D printed model of cervix (multiparous)	1.56 €
Modeling silicone (for negative footprints)	160.95 €
Modeling silicone (for silicone copy of the 3D printed models)	64.80 €
Additional consumables	20.0 €
**Sum**	**263.11 €**

Takacs et. al. achieved impressive results concerning acceptance and learning effect for novice gynecological surgeons by using a rather simple simulator [20, 21]. Our novel simulator seems more realistic and enables more procedural steps of a dysplasia consultation. Yet it demands a higher invest in resources e.g. money and time for production. Consequently, the question must be raised, if this simulator is accepted by clinical users and if it can generate better learning effects than conventional simulators, thereby justifying the higher invest for production. We plan to examine this in future studies.

## Conclusion

The described novel simulator can improve the training of novice gynecological surgeons, enabling us to establish high-quality standardized teaching. It seems to be more realistic and offers more training-variation than conventional simulators. Thus, we believe to offer a valuable possibility of improving surgeons’ proficiency as well as patients’ safety.

## Data Availability

The used 3D-model is the property of the corresponding author.

## References

[1] Bray F, Ferlay J, Soerjomataram I, Siegel RL, Torre LA, Jemal A (2018). Global cancer statistics 2018: GLOBOCAN estimates of incidence and mortality worldwide for 36 cancers in 185 countries. CA Cancer J Clin.

[2] Kerschgens C MZ, Koch MC, Beckmann MW (2014) *S3-Leitlinie Diagnostik, Therapie und Nachsorge der Patientin mit Zervixkarzinom.* S3-Leitlinie, Version 1.0 – September 2014, 2014. (**AWMF-Registernummer 032/033OL**)

[3] Bujan Rivera J, Sj Klug (2018). Bundesgesundheitsblatt Gesundheitsforschung Gesundheitsschutz. Cervical Cancer Screen Ger.

[4] Bornstein J, Bentley J, Bösze P, Girardi F, Haefner H, Menton M, Perrotta M, Prendiville W, Russell P, Sideri M, Strander B, Tatti S, Torne A, Walker P (2012). 2011 colposcopic terminology of the International Federation for Cervical Pathology and Colposcopy. Obstet Gynecol.

[5] Catarino R, Schäfer S, Vassilakos P, Petignat P, Arbyn M (2018). Accuracy of combinations of visual inspection using acetic acid or lugol iodine to detect cervical precancer: a meta-analysis. BJOG.

[6] Arbyn M, Redman CWE, Verdoodt F, Kyrgiou M, Tzafetas M, Ghaem-Maghami S, Petry KU, Leeson S, Bergeron C, Nieminen P, Gondry J, Reich O, Moss EL (2017). Incomplete excision of cervical precancer as a predictor of treatment failure: a systematic review and meta-analysis. Lancet Oncol.

[7] Kawano K, Tsuda N, Nishio S, Yonemoto K, Tasaki K, Tasaki R, Ushijima K (2016). Identification of appropriate cone length to avoid positive cone margin in high grade cervical intraepithelial neoplasia. J Gynecol Oncol.

[8] Papoutsis D, Rodolakis A, Mesogitis S, Sotiropoulou M, Antsaklis A (2013). Appropriate cone dimensions to achieve negative excision margins after large loop excision of transformation zone in the uterine cervix for cervical intraepithelial neoplasia. Gynecol Obstet Invest.

[9] Ghaem-Maghami S, De-Silva D, Tipples M, Lam S, Perryman K, Soutter W (2011). Determinants of success in treating cervical intraepithelial neoplasia. BJOG.

[10] Jeronimo J, Castle PE, Temin S, Denny L, Gupta V, Kim JJ, Luciani S, Murokora D, Ngoma T, Qiao Y, Quinn M, Sankaranarayanan R, Sasieni P, Schmeler KM, Shastri SS (2017). Secondary prevention of cervical cancer: ASCO Resource-Stratified Clinical Practice Guideline. J Glob Oncol.

[11] Santesso N, Mustafa RA, Wiercioch W, Kehar R, Gandhi S, Chen Y, Cheung A, Hopkins J, Khatib R, Ma B, Mustafa AA, Lloyd N, Wu D, Broutet N, Schunemann HJ (2016). Systematic reviews and meta-analyses of benefits and harms of cryotherapy, LEEP, and cold knife conization to treat cervical intraepithelial neoplasia. Int J Gynaecol Obstet.

[12] El-Nashar SA, Shazly SA, Hopkins MR, Bakkum-Gamez JN, Famuyide AO (2017). Loop electrosurgical excision procedure instead of cold-knife conization for cervical intraepithelial neoplasia in women with unsatisfactory colposcopic examinations: a systematic review and meta-analysis. J Low Genit Tract Dis.

[13] Liu Y, Qiu HF, Tang Y, Chen J, Lv J (2014). Pregnancy outcome after the treatment of loop electrosurgical excision procedure or cold-knife conization for cervical intraepithelial neoplasia. Gynecol Obstet Invest.

[14] Baldauf JJ, Dreyfus M, Wertz JP, Cuenin C, Ritter J, Philippe E (1997). Consequences and treatment of cervical stenoses after laser conization or loop electrosurgical excision. J Gynecol Obstet Biol Reprod (Paris).

[15] Castañon A, Landy R, Brocklehurst P, Evans H, Peebles D, Singh N, Walker P, Patnick J, Sasieni P (2015). Is the increased risk of preterm birth following excision for cervical intraepithelial neoplasia restricted to the first birth post treatment?. BJOG.

[16] Khalid S, Dimitriou E, Conroy R, Paraskevaidis E, Kyrgiou M, Harrity C, Arbyn M, Prendiville W (2012). The thickness and volume of LLETZ specimens can predict the relative risk of pregnancy-related morbidity. BJOG.

[17] Kyrgiou M, Athanasiou A, Kalliala IEJ, Paraskevaidi M, Mitra A, Martin-Hirsch PP, Arbyn M, Bennett P, Paraskevaidis E (2017). Obstetric outcomes after conservative treatment for cervical intraepithelial lesions and early invasive disease. Cochrane Database Syst Rev.

[18] Kyrgiou M, Athanasiou A, Paraskevaidi M, Mitra A, Kalliala I, Martin-Hirsch P, Arbyn M, Bennett P, Paraskevaidis E (2016). Adverse obstetric outcomes after local treatment for cervical preinvasive and early invasive disease according to cone depth: systematic review and meta-analysis. BMJ.

[19] Sparić R, Tinelli A, Guido M, Stefanović R, Babović I, Kesić V (2016). The role of surgeons' colposcopic experience in obtaining adequate samples by large loop excision of the transformation zone in women of reproductive Age. Geburtshilfe Frauenheilkd.

[20] Takacs FZ, Gerlinger C, Hamza A, Findeklee S, Juhasz-Boss I, Breitbach GP, Solomayer EF, Radosa JC (2020). A standardized simulation training program to type 1 loop electrosurgical excision of the transformation zone: a prospective observational study. Arch Gynecol Obstet.

[21] Takacs FZ, Radosa JC, Gerlinger C, Findeklee S, Juhasz-Boss I, Solomayer EF, Hamza A (2019). Introduction of a learning model for type 1 loop excision of the transformation zone of the uterine cervix in undergraduate medical students: a prospective cohort study. Arch Gynecol Obstet.

[22] Montanari E, Grimm C, Schwameis R, Kuessel L, Polterauer S, Paternostro C, Husslein H (2018). Influence of training level on cervical cone size and resection margin status at conization: a retrospective study. Arch Gynecol Obstet.

[23] Hefler L, Grimm C, Kueronya V, Tempfer C, Reinthaller A, Polterauer S (2012). A novel training model for the loop electrosurgical excision procedure: an innovative replica helped workshop participants improve their LEEP. Am J Obstet Gynecol.

[24] Rezniczek GA, Severin S, Hilal Z, Dogan A, Krentel H, Buerkle B, Tempfer CB (2017). Surgical performance of large loop excision of the transformation zone in a training model: A prospective cohort study. Medicine (Baltimore).

[25] Seltzer MS, Habermehl DA, Julian TM (1997). A comparison of loop electrosurgical excision, laser ablation, and cold-knife conization in relation to precise specimen removal in an inanimate model. J Low Genit Tract Dis.

[26] Wilson EB, Beckmann MM, Hewett DG, Jolly BC, Janssens S (2017). Evaluation of a low-fidelity surgical simulator for large loop excision of the transformation zone (LLETZ). Simul Healthc.

[27] Reeves KO, Young AE, Kaufman RH (1999). A simple, inexpensive device for teaching the loop electrosurgical excision procedure. Obstet Gynecol.

[28] Connor RS, Dizon AM, Kimball KJ (2014). Loop electrosurgical excision procedure: an effective, inexpensive, and durable teaching model. Am J Obstet Gynecol.

[29] Manley KM, Park CH, Medland VL, Appleyard TL (2015). The training value of a low-fidelity cervical biopsy workshop. Simul Healthc.

[30] Vella PV (2002). A simple trainer for the loop electrosurgical excision procedure. Aust N Z J Obstet Gynaecol.

[31] Walters CL, Whitworth JM, Tyra SL, Walsh-Covarrubias JB, Straughn JM (2013). Constructing a novel simple LEEP training model. J Grad Med Educ.

[32] Russomano, F (2018) *LLETZ cone or type 3 excision of the transformation zone*. [Online Video] 2015 [cited 2020 30.05.2020]; Demonstrational video of LLETZ-procedure]. https://www.youtube.com/watch?v=8xSp0QuC7XU. Accessed 30 May 2020

[33] Gross_Cutting_Room (2020) *Gross Cutting Room | Episode: 16 | Cervical Cone Dissection*. 30.05.2020]; Grossing tutorial of a cervical cone]. https://www.youtube.com/watch?v=dLMFEWMEyNo. Accessed 30 May 2020

